# Controversial Topics in Total Knee Arthroplasty: A 5-Year Update (Part 1)

**DOI:** 10.5435/JAAOSGlobal-D-19-00047

**Published:** 2020-01-03

**Authors:** Johannes Michiel van der Merwe, Matthew Semrau Mastel

**Affiliations:** From the Department of Orthopaedics, University of Saskatchewan, Saskatoon, Saskatchewan, Canada.

## Abstract

This review article examines updates to the literature during the past 5 years on numerous topics related to total knee arthroplasty which were felt to have ongoing controversy. These include the use of peripheral nerve blocks and local infiltrative analgesia, intrathecal morphine, patellar resurfacing, and bearing designs.

**Methods:**

For each individual topic, a literature search was conducted on several databases with emphasis on studies that were published in the past 5 years. Preference was given to meta-analyses and randomized controlled trials.

**Results:**

Multimodal periarticular injections may provide an equally effective analgesic effect to peripheral nerve blocks, but are also muscle sparing and less invasive. The use of intrathecal morphine in addition to periarticular injections is less desirable given the potential side effects, associated cost, and lack of clear benefit intrathecal morphine beyond the 6- to 12-hour postoperative period. Patellar resurfacing was associated with a lower rate of revision surgery, similar or potentially improved satisfaction and functional outcomes, and no increased risk of complications compared with nonresurfacing. There are no clear or notable differences between cruciate-retaining and posterior-stabilized total knee designs in terms of clinical outcomes and survivorship. Medial pivot designs theoretically recreate more normal knee kinematics compared with cruciate-retaining or posterior-stabilized designs, although superiority has not yet been clearly demonstrated and additional long-term data is necessary, particularly for survivorship.

**Conclusions:**

By analyzing the results of the aforementioned studies, surgeons can implement the most up-to-date evidence-based care when doing total knee arthroplasty surgery. However, many of these selected topics continue to have a component of ongoing controversy with no definitive conclusions developed in recent literature.

Total knee arthroplasty (TKA) is a very commonly done orthopaedic procedure, and therefore, any improvements in technique may have a notable effect on the patient cohort. Despite the frequency of TKA, there are notable variations in techniques with many controversies existing. This review article examines updates to the literature during the past 5 years on numerous topics which were felt to have ongoing controversy or discrepancies between the techniques used by orthopaedic surgeons. In this particular article, attention is focused on the use of peripheral nerve blocks and local infiltrative analgesia, intrathecal morphine, patellar resurfacing, and bearing designs. Pain control in TKA has become an important issue because it affects functional recovery and the overall success of the knee replacement.^1^ As a result, multiple analgesia modalities have gained increased popularity including peripheral nerve blocks, local infiltrative analgesia, and epidural or intrathecal analgesia. However, each option presents with benefits and limitations, and the method of utilization is often variable.^2,3,4,5,6^ Patellar resurfacing has remained a controversial issue in TKA. Not resurfacing may lead to increased rates of anterior knee pain, along with higher reoperation rates for subsequent patellar resurfacing. Alternatively, resurfacing may lead to increased patellar related complications such as fracture or instability, component wear/loosening, osteonecrosis, and patellar tendon injury.^23^ There has been a long-standing debate regarding the superiority of cruciate-retaining (CR) or posterior-stabilized (PS) cruciate-substituting implants with advantages and disadvantages to each. Recently, greater interest has been paid to recreating normal knee kinematics, with increased attention on medial pivot designs. These components place emphasis on reproducing posterior femoral roll back of the lateral compartment while maintaining a pivot motion medially.^36^ This review was not intended to be a comprehensive review of all these specific topics, but rather to be a compilation overview of updates to the literature from the past 5 years. By analyzing the results of recent studies, we can implement the most up-to-date and evidence-based care for our patients when performing TKA surgery.

## Methods

For this review, four topics were selected that were felt to have ongoing controversy among orthopaedic surgeons and in the literature. These topics include peripheral nerve blocks and local infiltrative analgesia, intrathecal morphine, patellar resurfacing, and bearing designs (CR/PS/medial pivot). For each individual topic, a literature search was conducted on several databases that included but was not limited to PubMed, the University of Saskatchewan Online Library Catalogue, the *Journal of the American Academy of Orthopaedic Surgeons*, and Google Scholar. Searches were conducted using a variety of search terms related to each specific topic in addition to the following terms: TKA, total knee, and arthroplasty. As the focus of this study was to examine updates to the literature, emphasis was placed on studies that were published in the past 5 years (2014 to 2018 inclusive), with exceptions made in the case to provide background information on a topic, or studies that contain the most recently available data. Preference was given to meta-analyses, randomized controlled trials (RCTs), and commonly used guideline sources although other studies of lower level of evidence were included as required. Studies of the highest quality of evidence or most relevance were given priority. Only articles and guidelines written in English that were available in full-text format were included. For topics with numerous studies available from the past 5 years, the outcomes of each study were compiled into a table format.

## Adductor Canal Blocks, Femoral Nerve Blocks, and Local Infiltrative Analgesia

Pain control in TKA has become a very important issue. Uncontrollable pain after a TKA is one factor which can affect functional recovery and the overall success of the knee replacement.^1^ Extreme postoperative pain can lead to excessive opioid consumption with opioid-related side effects, including respiratory depression. Proper analgesia can also reduce chronic postsurgical pain that can last more than 6 months after surgery. Multiple treatment modalities have been introduced to control pain better after a replacement, which includes ischemic preconditioning, peripheral nerve blocks, epidural analgesia, local infiltrative analgesia, and patient-controlled opioids. However, there are limitations to each modality (patient-controlled opioids: nausea and vomiting; epidural analgesia: urinary retention and pruritus; peripheral nerve blocks: decrease muscle strength; local infiltration: short-term action) and the optimal method of pain control after a knee replacement is still unknown.

This review article specifically looked at the most commonly used options for pain management postoperatively. These options include adductor canal blocks (ACBs); femoral nerve blocks (FNBs), multimodal periarticular infiltrations (MPIs); combinations of nerve blocks (ACBs, FNBs, obturator nerve blocks, and sciatic nerve blocks); and field blocks (fascia iliaca blocks and lumbar plexus blocks).

The adductor canal is an aponeurotic tunnel in the middle of the thigh. It contains the vastus medialis nerve, the medial femoral cutaneous nerve, articular branches from the obturator nerve, the medial retinacular nerve, and the saphenous nerve. It is almost a pure sensory nerve block but does affect the motor function of the vastus medialis. By blocking these nerves in the adductor canal, one can achieve pain relief after a TKA.^2,3^ It is usually done through ultrasonography guidance which is used to identify the sartorius muscle and the femoral artery. The leg will be slightly flexed and externally rotated during the nerve blockade. The adductor canal is entered by traversing the sartorius muscle. Once the needle is in the correct spot, then 5 to 10 mL of bupivacaine or ropivacaine is injected into the canal.

FNB technique involves identification of the femoral nerve below the inguinal ligament using nerve stimulation and/or ultrasonography, followed by infiltration of levobupivacaine hydrochloride around the femoral nerve.

MPIs work by injecting a solution consisting of an opioid (2 to 5 mg of morphine), anti-inflammatory (30 to 40 mg of ketorolac), epinephrine (1:200,000 parts epinephrine), a local anesthetic (30 to 40 mL of 0.25% ropivacaine), and 30 to 60 mL of distilled water periarticularly at the time of the knee replacement.^3,4,6^ There is a variation in contents and methods of injection. For instance, periarticular injections can occur partly before implantation of the implants or after implantation. Various techniques include injecting the posterior and anterior capsule, medial and lateral collateral ligaments, retinacular tissues, muscles and subcutaneous tissues, or a combination thereof.^2,3,4,5,6^

Recently, the addition of an ACB to the MPI has gained interest by both the anesthesiologists and the orthopaedic surgeons. In theory, by combining the two modalities (MPI and ACB), one would expect to see longer pain relief with less overall opioid consumption and slightly weaker quadriceps function compared with MPI alone. When we look at all the evidence comparing these two modalities (MPI alone versus MPI combined with ACB) (Table 1), the majority of the evidence does not show any difference.^3,4,5,6^ Only one study showed a positive difference in the combined MPI and ACB.^2^ Therefore, there might be a benefit in early postoperative pain control with the combined MPI and ACB with potentially slightly weaker quadriceps muscle function due to the ACB.

**Table 1:** The Most Recent Evidence for Postoperative Pain Control in Total Knee Arthroplasty Comparing ACB Combined With MPI (Combined) Against MPI; FNB Against MPI; and FNB Against ACB

Study and Comparison	Pain POD 0	Pain POD 1	Pain POD 2	Nausea and Vomiting	Length of Hospital Stay	Morphine Consumption
**ACB + MPI (Combined) versus MPI or ACB**						
Xing et al^2^—total 297 patients (combined 149; MPI 148): meta-analysis	Better pain control: (combined) (*p* = 0.001)	Better pain control (combined) (*P* = 0.00)	Better pain control (combined) (*P* = 0.007)	Less nausea and vomiting (combined) (*P* = 0.024)	No difference (*P* = 0.120)	Lower opioid consumption (combined) POD 0, 1, 2 (*P* = 0.006)
Ma et al^3^—total 337 patients (combined 171; MPI 166): meta-analysis	No difference (*P* = 0.41)	No difference (*P* = 0.91)	No difference (*P* = 0.06)	No difference (*P* = 0.69)	No difference (*P* = 1.00)	No difference (*P* = 0.07)
Sankineani et al^4^—total 200 patients (combined 100, ACB 100): nonrandomized study	Better pain control (combined) (*P* = 0.0212)	No difference	No difference	Not recorded	Not recorded	Not recorded
Gwam et al^5^—total 110 patients (combined 65; MPI 45): retrospective review	Not reported	Not reported	Not reported	Not reported	No difference (*P* = 0.304)	No difference (*P* = 0.729)
Gwam et al^6^—total 127 patients (ACB 52; combined 75): retrospective review	Not reported	Not reported	Not reported	Not reported	No difference (*P* = 0.934)	No difference (*P* = 0.708)
**FNB versus MPI**						
Ma et al^7^—total 1,289 patients (FNB and MPI): meta-analysis	No difference (*P* = 0.688)	No difference (*P* = 0.749)	No difference (*P* = 0.575)	No difference (*P* = 0.914)	Shorter length of stay (MPI) (*P* < 0.0001)	Lower morphine consumption POD 1 + 2 (MPI) (*P* = 0.037)
Liu et al^8^—total 2,407 patients (FNB 1,293) and (MPI 1114): meta-analysis	No difference (*P* = 0.284)	No difference (*P* = 0.076)	No difference (*P* = 0.795)	No difference (*P* = 0.617)	Shorter length of stay (MPI) (*P* = 0.001)	No difference (*P* = 0.042)
Wall et al^9^—total 262 patients (FNB 131; MPI 131): RCT	Not reported	No difference (*P* = 0.770)	No difference (*P* = 0.435)	No difference	Not reported	Less morphine consumption up to 24 hr in favour FNB (*P* = 0.042)
Fan et al^10^—total 157 (FNB 78; MPI 79): randomized double-blinded single-center study	Not reported	No difference (*P* = 0.78)	No difference (*P* = 0.11)	No difference	Not reported	No difference (*P* = 0.18)
Wang et al^11^—total 744 total knee replacements in 728 patients: meta-analysis	Better pain control (FNB) (*P* = 0.001)	No difference (*P* = 0.62)	No difference (*P* = 0.70)	No difference (*P* = 0.93)	Shorter hospital stay in favour MPI (*P* = 0.04)	No difference at 24 hr or 48 hr (*P* = 0.64; *P* = 0.99)
**FNB versus ACB**						
Kuang et al^12^—total 609 patients: meta-analysis	No difference (*P* = 0.21)	No difference (*P* = 0.30)	No difference (*P* = 0.18)	Not reported	No difference (*P* = 0.42)	No difference POD 0 (*P* = 0.45); POD 1 (*P* = 0.96); POD 2 (*P* = 0.15)
Macrinici et al^13^—98 patients (ACB 49; FNB 49): RCT	No difference	No difference	No difference	Not reported	No difference	No difference
Wiesmann et al^14^—42 patients (ACB 21; FNB 21): RCT	Better pain control (ACB) (*P* = 0.04)	No difference (*P* = 0.99)	No difference (*P* = 0.57)	Not reported	Not reported	No difference
Machi et al^15^—80 patients (ACB 39; FNB 41): RCT	No difference (*P* = 0.864)	No difference (*P* = 0.876)	No difference (*P* = 0.562)	Not reported	No difference	No difference (*P* = 0.480)

ACB = adductor canal block, FNB = femoral nerve block, MPI = multimodal periarticular infiltration, RCT = randomized controlled trial; POD = post-operative day

FNBs provide the benefit of longer lasting analgesia although at the cost of greater quadriceps dysfunction and theoretically longer hospital stay. When we look at the evidence (Table 1) comparing FNBs to MPIs, it is evident that periarticular infiltrations are as good as FNBs to control pain postoperatively with the added benefit of not impairing the quadriceps function which could lead to shorter hospital stays.^7,8,9,10,11^

FNBs compared with ACBs do not show any difference in terms of pain control, total opioid consumption, or earlier discharge.^12,13,14,15^ There is some evidence that FNBs do offer superior dynamic analgesia (pain control with passive movement) but with the caveat of dysfunction of the quadriceps musculature.^11^ To determine the optimal choice, one must consider the goal they are trying to achieve. For a patient with chronic pain issues, or on long-term narcotics where acceptable pain control is a potential issue, a continuous FNB might be a good option. If earlier mobilization is the goal, then an adductor nerve block or a continuous ACB might be a more suitable option.

There is some evidence supporting combining multiple blocks for pain control.^1^ Although this is still a very controversial topic with studies confirming and refuting the benefits of combining blocks, some argue that a FNB combined with a sciatic nerve block decreases pain more than a single nerve block due to the analgesia effect on the posterior knee capsule.^1^ Lumbar plexus blocks were associated with more block failures than other modalities, which demonstrated their difficulty to do. The lumbar plexus block only showed notable benefit when combined with a sciatic nerve block, which highlighted the importance of posterior capsule analgesia. Fascia iliaca blocks have very limited evidence to support their usage routinely in TKA.

In summary: There are multiple modalities available to control pain postoperatively after a knee replacement. The reason for the existence of these numerous modalities is that there is no single modality fulfilling all the requirements for pain control after a TKA. As there are only very small differences in the efficacy of these numerous modalities, one should use the least invasive modality that will lead to a quick and satisfactory recovery. We prefer to use multimodal periarticular injections. It not only provides an equally effective analgesic effect but also is muscle sparing and is less invasive than peripheral nerve blocks.

## Intrathecal Morphine in Total Knee Arthroplasty

The addition of intrathecal morphine (ITM) during a spinal anesthetic theoretically produces more effective and longer term anesthesia. ITM does have side effects that are dose dependent which include itching, nausea, and vomiting. The usual administration occurs at the time of the spinal anesthetic. The patient is positioned in a sitting position, and the back is properly prepped with an alcohol-based solution. Once the spinal needle is inserted and a backflow of cerebrospinal fluid is observed, then the bupivacaine is injected followed by morphine. The smallest effective dose of morphine with the least amount of adverse effects is 100 micrograms. Multiple RCTs have evaluated the efficacy and adverse effect profile with the addition of ITM. Pain is reduced with the addition of ITM in the first several hours after a knee replacement. Two studies demonstrated notable clinical improvement in pain at 6 hours^16^ and 12 hours^17,18^ after surgery compared with isolated multimodal periarticular infiltration. However, there was also a notable increase in side effects (such as itching and urinary retention) associated with ITM administration. Interestingly, nausea and vomiting was not a notable symptom, even with larger morphine doses.^16,19^

Urinary retention secondary to ITM has recently resulted in a shift away from using ITM during a spinal anesthetic despite its better analgesia effect. Urinary catheterization for urinary retention is a troublesome complication because of threefold increase in deep infection after a replacement.^20,21^ Some studies have demonstrated an increased risk of urinary retention with ITM in older male patients.^20,21^ Not only did the use of ITM lead to urinary retention but it also caused an increased length of stay in the affected individuals.

In summary: The use of ITM may provide improved pain control during the first 6 to 12 hours after a TKA. However, potential side effects such as itching and urinary retention, along with the increased cost associated with ITM, and no clear benefit compared with multimodal periarticular infiltration after 6 to 12 hours following a TKA make the practicality of ITM less desirable. Low dosage ITM may be a good addition in elderly patients at risk of serious opioid side effects knowing that this cohort group is at risk of urinary retention and its sequelae.

## Patellar Resurfacing

Patellar resurfacing has remained a controversial issue in TKA with anterior knee pain being the most commonly reported symptom after TKA regardless whether patellar resurfacing was performed or not.^22^ Initial TKAs were implanted without resurfacing the patella; however, because of the prevalence of anterior knee pain, the tricompartmental TKA was designed. This addition had led to an increase in complications related to the patella,^22^ with controversy still remaining around the best option for patients. Not resurfacing can lead to increased rates of anterior knee pain, along with reoperation for patellar resurfacing with the assumption the source of pain was the unsurfaced patella. Alternatively, it has been suggested resurfacing may lead to complications including patellar fracture or instability, component wear/loosening, osteonecrosis, and patellar tendon injury.^23^ However, with the improvement of techniques and implant design, the risk of these complications has been markedly diminished.

There are several meta-analyses published since 2011 with the general consensus being patellar resurfacing reduces the risk of reoperations, but no conclusions have been made regarding the benefit of resurfacing on anterior knee pain or function.^24^ This uncertainty has led surgeons to follow three main approaches to the patella: always resurface, never resurface, or selectively resurface based on patient specific factors.^22^ Some recommendations suggest that patellar retention can be done in select patients who are younger than 60 years, have minimal cartilage degeneration, with a well-contoured patella that tracks well. Alternatively, other studies have demonstrated success with routine patellar resurfacing. Numerous studies place emphasis on selectivity, with factors such as body mass index, weight, patellar alignment, and degree of chondromalacia factoring into the decision for patellar management.^25^ Unfortunately, the evidence does not support any one approach over the others, and there continues to be a lack of clear guidelines regarding patellar resurfacing.^25^

Pilling et al^22^ have done a meta-analysis involving 3,465 TKAs, 1,755 with and 1,710 without resurfacing. They concluded there was no significant difference in patient satisfaction (90.0% resurfaced and 89.1% nonresurfaced), anterior knee pain (13.4% in resurfaced and 23.5% nonresurfaced; *P* = 0.1), or infection rates. Only the Knee Society Score (KSS) demonstrated a statistical difference in favor of resurfacing, although the absolute difference was felt to be clinically insignificant. Reoperations for anterior knee pain were greater in the nonresurfaced group (6% versus 1%, *P* < 0.00001), while there was no difference in rates of patellofemoral complications when anterior knee pain as the only report was eliminated (1.3% resurfaced and 1.1% nonresurfaced; *P* = 0.62). There was no notable differences in surgical time between the procedures. Overall, they suggested the decision must be made by the surgeon and patient together, with more evidence required to make firm conclusions.

The most recent meta-analysis, to the best of our knowledge, by Chen et al^23^ produced similar results. They analyzed 14 RCTs with a total of 1,725 TKAs. They determined the relative risk of reoperation favored resurfacing (relative risk [RR] 0.50, *P* = 0.001) with the absolute risk of reoperation being reduced by 4%. Overall, there was no difference in terms of anterior knee pain (*P* = 0.46, significant heterogeneity), but they did suggest that during long-term follow-up of 5 or more years, the resurfaced group may have higher KSSs therefore favoring resurfacing. However, they felt more RCTs of greater quality would be required to support this.

One of the concerns that has been raised when analyzing anterior knee pain and knee function is the ability of classic outcome measures such as the Knee Society clinical rating system to discriminate properly due to high ceiling effects.^24^ Recently, Steinhoff and Bugbee^24^ suggested that the Knee Injury and Osteoarthritis Outcome score (KOOS) has greater responsiveness and lower ceiling effect compared with the Knee Society Function score making it more optimal for evaluation of TKA patients. With these considerations, Aunan et al^26^ have done a double-blind RCT of 129 TKAs with 3-year follow-up, with their primary outcome measure the KOOS, being in favor of patellar resurfacing. The greatest difference was seen at 3 years in the subscore sport/recreation section with a 10-point difference (*P* = 0.01), with the next greatest difference being eight points for the knee-related quality of life subscore (0.03). However, the minimal perceptible clinical improvement for KOOS was suggested by Roos and Lohmander^41^ to be 8 to 10 points. Therefore, the clinical significance is still unclear. However, there was no differences in secondary outcomes (KSS function score, Oxford knee score, or patient satisfaction).^24^

Other recent results from randomized control trials during the past 5 years appear to demonstrate minimal differences between resurfacing and retention in terms of anterior knee pain, complications, and traditional knee scores (Table 2).^26,27,28,29^ A RCT by Koh et al^27^ involved 49 patients receiving bilateral TKAs with one knee randomized to be resurfaced revealed no differences in anterior knee pain, Forgotten Joint Score, Feller patellofemoral score, or side preference (all not statistically significant). One study demonstrated a notable difference in patient satisfaction favoring resurfacing,^29^ while the previously mentioned study by Aunan et al suggested a functional benefit to resurfacing.^24^ However, the clinical significance of these results is still unknown.

**Table 2:** Recent Studies Comparing Patellar Resurfacing and Non-Resurfacing

	Patients	Follow-up	Results	Complications	Recommendation
Koh et al^27^: RCT	49 receiving bilateral TKA (1 side randomized to RS)	Mean 5 yr	No difference in anterior knee pain (2.8% RS, 1.8% NR), Forgotten Joint Score, Feller patellofemoral score, or side preference; all not significant	No RS-related complications in (RS)	Patients unaware of differences with RS
				No secondary RSs in (NR)	No increased risk of reoperation regardless of option
Ali et al^28^: RCT	74 using triathlon; CR TKA	6 yr	No difference in VAS pain, patient satisfaction, or KOOS at 3, 12, and 72 mo	No secondary RSs in (NR)	Patella RS unnecessary
Aunan et al^26^: single center, double blind RCT	129 TKAs (115 patients)	3 yr	Mean KOOS subscores statistically significantly in favor of (RS) (sport/recreation 10-pt difference, knee-related quality-of-life 8-pt difference, pain 6-pt difference, and symptoms 5-pt difference; all *P* < 0.05)	Three complications in (NR) group (one patella fracture, one patient with stiffness, one partial quads rupture)	KOOS indicated (RS) may be beneficial for knee function, while traditional outcome measures failed to demonstrate a difference
			No difference with Knee Society clinical rating system, VAS, oxford knee scores, or patient satisfaction	Four complications in (RS) group (one patient with stiffness, one patient with lateral knee pain and stiffness, and one hematogenous infection 2 yr later)	
Roberts et al^29^: double blind RCT	327 TKAs (255 patients)	Mean 7.8 yr	Satisfaction lower if (NR) (*P* = 0.039), although not significant in subset with 10 yr f/u	14 revisions total: rate = 5.8% (NR), 2.8% (RS) at 10 yr (*P* = 0.20)	Statistical difference in patient satisfaction at final f/u favoring (RS), but clinical significance may be minimal
	Subset 114 TKAs (88 patients)	Mean 10.4 yr	No difference in Knee Society scores, ROM	4 revisions for anterior knee pain (all [NR]), remaining revisions done for chronic effusions/synovitis	No complications specific to patellar RS up to 12 yr f/u
			No difference in anterior knee pain with walking (2.1% [NR], 3.0% [RS]; *P* = 1.00)		Can confidently resurface without risks previously associated with earlier implants

CR = cruciate-retaining, KOOS = Knee Injury and Osteoarthritis Outcome score, NR = nonresurfacing, RCT = randomized controlled trial, RS = resurfacing, TKA = total knee arthroplasty; VAS = visual analog scale; ROM = range of motion

In summary: There continues to be a lack of clear guidelines regarding patellar resurfacing. Anterior knee pain is the most common presenting report after TKA with or without patella resurfacing. Although absolute rates of anterior knee pain tend to be higher without resurfacing, the differences have not been shown to be statistically significant. However, the presence of anterior knee pain in the nonresurfacing group leads to markedly more reoperations with no guaranteed improvement of symptoms thereafter. There is also some evidence suggesting better functional recovery with patella resurfacing. Despite these findings, the evidence is still lacking in clinical significance. Therefore, it is reasonable for surgeons to continue using their current strategy (resurface all, resurface none, or selectively resurface) until additional evidence emerges. Given the lowered rate of revision surgery with resurfacing, along with similar or potentially improved satisfaction and functional outcomes, while at no increased risk of complications, our preference is to resurface all patellas.

## Total Knee Designs

There has been a long-standing debate regarding the superiority of cruciate retaining or substituting implants.

**Figure 1:**
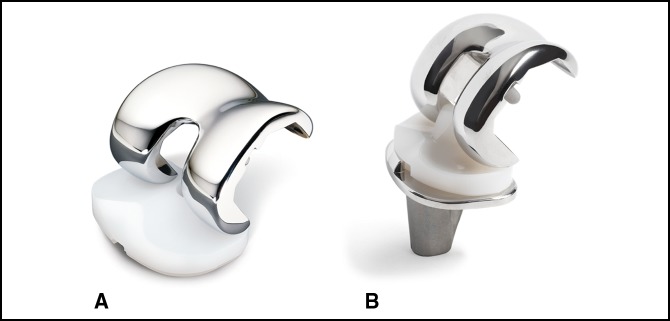
Photographs of Zimmer Nexgen components demonstrating the difference between the cruciate-retaining design (**A**) and the posterior-stabilized design (**B**) which incorporates a central tibial post and femoral cam to simulate the posterior cruciate ligament. Reproduced with permission from Zimmer Biomet, January 2019.

CR components (Figure 1, A) rely on an intact PCL to limit posterior translation of the tibia and require appropriate balancing of the PCL in flexion and extension. Many benefits of CR components have been proposed including retention of proprioceptive fibers and more normal knee kinematics, improved quadriceps strength and stair-climbing ability, decreased shear forces at bone-component interface, less femoral bone resection, and avoidance of dislocation over the tibial post that can occur in PS components.^30^ However CR components do have several potential disadvantages as well. Some studies have shown retention of the PCL may reduce the naturally occurring femoral roll-back of the distal femur on the tibia with knee flexion when improperly balanced.^31^ In addition, there is a potential risk of PCL rupture postoperatively which may be attributable to excessive intraoperative PCL recession, overtightening from an altered joint line, or damage resultant from synovitis.^30^

PS components (Figure 1, B) incorporate design features such as a tibial post and femoral cam or deep dished articular surfaces to simulate the PCL and limit tibial translation. Numerous benefits exist to PS implants including the relative ease of ligament balancing, greater versatility in differing knee deformities, more predictable restoration of knee kinematics, and potentially less polyethylene with the use of more congruent articular surfaces. However, the cam-post mechanism can also cause excessive polyethylene wear leading to osteolysis. Soft-tissue impingement can occur when soft-tissue nodules catch in the intracondylar notch during knee flexion causing the painful patellar “clunk” syndrome. Other disadvantages include greater bone resection and the possible risk of dislocation.^30^

A 2013 Cochrane review examined 17 RCTs with a total of 1,810 patients and 2,206 TKAs. Range of motion (ROM) was found to be 3.4° higher in PS implants (*P* = 0.02). The mean functional KSS was 2.3 points greater in the PCL-sacrificing group (*P* = 0.02). However, both of these statistically notable findings were believed to be clinically irrelevant. Meta-analysis was done on the Knee Society knee pain scores, which were 48 for both groups (no statistical difference). In addition, the Western Ontario and McMaster Universities Osteoarthritis Index (WOMAC) total score showed no statistically notable difference between groups (16.6 points cruciate-retention versus 18.2 points cruciate-sacrifice). All remaining outcome measures including knee pain, extension angle, clinical questionnaire scores, Knee Society clinical scores, radiologic roll back, femoral-tibial angle, tibial slope, and radiolucencies showed no statistically notable differences. Complications were equally distributed between the groups. The quality of evidence of the articles ranged from moderate to low; however, the authors concluded there was no clinically relevant differences between retention and sacrifice of the PCL with respect to ROM, pain, clinical, and radiologic outcomes.^31^

Several studies have looked at the survivorship of CR and cruciate-substituting implants. Abdel et al^32^ had done a retrospective review of 8,117 TKAs from 1988 to 1998 demonstrating a significant benefit in survival with CR components (15-year survival rates: 90% for CR and 77% for PS designs [*P* < 0.001]). Alternatively, Sando et al^33^ compared 414 CR versus PS TKAs using the Genesis II knee system and found excellent survival rates with no significant difference in 10-year survivorship (98.6% for CR versus 96.5% for PS, *P* = 0.22). Li et al^34^ have done a systematic review and meta-analysis that included eight RCTs with 888 patients and 963 TKAs comparing clinical efficacy and prosthesis survivorship of the two TKA designs. There was no notable difference in survivorship when assessing longer follow-up studies (CR 90% to 98% at 9 to 10 years, PS 92% to 98% at 10 to 16 years). There were no differences in knee society pain scores, 2- and 5-year KSSs, or 2- and 5-year Knee Society Function scores. There were no statistical difference rates of postoperative complications including anterior knee pain, infection, deep vein thrombosis, or revision arthroplasty. However, when assessing postoperative ROM, the PS designs had 11.07° higher ROM than CR (*P* < 0.01). Kremers et al^35^ have done a retrospective review of 12,482 patients undergoing 17,192 primary TKAs at Mayo Clinic using 40 different tibial implants from 1985 to 2005. With the exception of the Johnson & Johnson PFC (Press Fit Condylar) component which had design failure leading to accelerated wear, there was no difference in survivorship between CR and PS knees for both metal-backed and all-polyethylene components. The overall survivorship was 94% at 10 years and 88% at 15 years. They also reported that obese patients (body mass index > 35) had higher rates of failure requiring revision with CR components (Hazard Ratio, 1.3; 95% confidence interval (95% CI), 1.0 to 1.6) compared with PS designs where no difference was seen. Given the advances in component designs and manufacturing, newer studies would be beneficial to determine current differences in survivorship.

### Medial Pivot Designs

**Figure 2:**
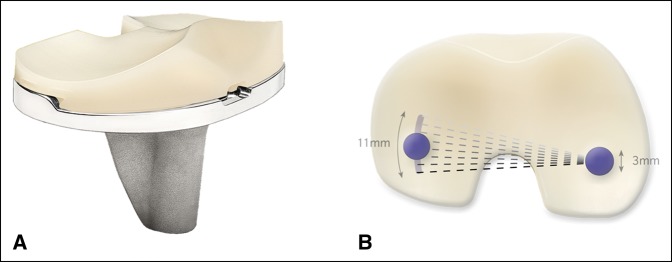
Photograph of the Zimmer Persona Medial Congruent Bearing (**A**) and diagram of the pivoting motion (**B**) where the medial femoral condyle remains more stationary as the lateral condyle is allowed anterior-posterior translation along a 14° arcuate path. Reproduced with permission from Zimmer Biomet, January 2019.

Recently, greater interest has been paid to recreating normal knee kinematics, with increased attention on medial pivot designs. Studies of normal knee kinematics demonstrate that the medial compartment of the knee is more stable and congruent while the lateral compartment translates anterior and posterior and “pivots” around the medial compartment. Therefore, an emphasis has been placed on reproducing both posterior femoral roll back of the lateral compartment while maintaining a medial pivot motion. It is felt that the main advantage of medial pivot designs (Figure 2) is increased contact area and improved kinematics rather than improved knee flexion. It was suggested that patients with different bilateral TKA components tend to prefer the medial pivot implant over the contralateral CR or PS design citing the improved stability and more normal feel.^36^ A retrospective analysis of 150 medial pivot TKAs done in 125 patients was compared with a control group with PS designs. There was satisfactory functional and pain recovery in both groups, with no notable difference between the two groups in terms of ROM, Knee Society's Knee Scoring System, WOMAC, or Kujala and Feller scoring systems.^37^ Fitch et al^38^ have done a systematic review and meta-analysis that included eight studies with 1,146 TKAs using the ADVANCE Medial-Pivot System. They suggested that pooled survivorship of medial pivot designs was markedly greater than results reported by the National Joint Registry of England, Wales, and Northern Ireland for unconstrained mobile bearings, PS mobile bearings, and constrained condylar bearings at 4, 5, and 6 years as well as PS fixed bearings at 6 years. Overall, the survivorship was found to be 99.2% at 5 years and 97.6% at 8 years. The revision rate due to instability, insert exchanges, or insert breakage was 0.26%, and they suggested there are no increased rates of failure caused by the increased constraint on the medial compartment of the implant. The weighted mean KSS was 87.9 which falls in the range of excellent (80 to 100), although this value is similar to that reported for various other types of TKA systems. A retrospective review of 506 TKAs also using the ADVANCE Medial-Pivot System found a 10-year survivorship of 96.3%, similar to other cemented TKA systems,^39^ while another retrospective review of 284 TKAs in 225 patients using the ADVANCE Medial-Pivot System found a 15-year survivorship of 97.3%.^40^ Midterm results of medial pivot designs are satisfactory with clinical scores and survivorship seemingly similar to other designs. However, more long-term data are necessary to make definitive conclusions.

In summary: There are no clear or notable clinical differences between CR and PS designs. Patients have similar clinical outcomes, and several studies have demonstrated similar survivorship. Medial pivot designs theoretically recreate more normal knee kinematics compared with CR or PS designs, and although an improvement in clinical outcomes has been suggested, it has not been clearly demonstrated. Patients do tend to prefer the medial pivot design in regard to a more normal feel, and the increased stability that it loans, although there is no clear evidence demonstrating superiority over other knee designs. The midterm survivorship of medial pivot designs has been similar to other designs, but additional long-term data are still necessary. Ultimately, given similar rates of patient satisfaction and component survivorship, the choice of component design should be based on the surgeon's preference, familiarity, and cost of the implants.

## Summary

There are numerous controversial topics in TKA, and four of these areas were addressed in this particular review article. Despite the evolution of literature specific to these topics, there continues to be an amount of uncertainty regarding the most beneficial and appropriate treatment choices. There are multiple analgesic modalities available to control pain postoperatively, and based on the recent literature, there are only very small differences between the efficacy of the various peripheral nerve blocks and multimodal periarticular infiltrations. Therefore, we prefer to use multimodal periarticular injections which provide an equally effective analgesic effect, but are also muscle sparing and less invasive than peripheral nerve blocks. The use of intrathecal morphine may provide improved pain control during the first 6 to 12 hours postoperatively. However, the potential side effects such as itching and urinary retention, along with the increased cost, and no clear benefit demonstrated compared with multimodal periarticular infiltration after 6 to 12 hours postoperatively make the practicality of ITM less desirable. There continues to be a lack of clear guidelines regarding patellar resurfacing. Given the lowered rate of revision surgery after resurfacing, along with similar or potentially improved satisfaction and functional outcomes, while at no increased risk of complications, our preference is toward patellar resurfacing. There are no clear or notable differences between CR and PS total knee designs in terms of clinical outcomes and survivorship. Medial pivot designs theoretically recreate more normal knee kinematics compared with CR or PS designs, and although an improvement in clinical outcomes has been suggested with patients tending to prefer the more normal feel, superiority has not yet been clearly demonstrated and additional long-term data is necessary, particularly for survivorship. These conclusions that we arrived at are based on studies done in the past 5 years and are certainly not a comprehensive overview of each of the topics. Orthopaedic surgeons should use these recommendations cautiously and only as an adjunct to their existing knowledge of the topic. There has been an abundance of literature published in the past 5 years attempting to address these problems with TKA, and future studies will only continue to improve the functional outcomes and satisfaction in our patients. By analyzing the results of the aforementioned studies, surgeons can implement the most up-to-date evidence-based care when doing TKA surgery.
